# Exosomal miR-184 in the aqueous humor of patients with central serous chorioretinopathy: a potential diagnostic and prognostic biomarker

**DOI:** 10.1186/s12951-023-02019-6

**Published:** 2023-07-28

**Authors:** Jee Myung Yang, Soo Jin Kim, Seongyeol Park, Wonyung Son, Anna Kim, Junyeop Lee

**Affiliations:** 1 Department of Ophthalmology, Asan Medical Center, College of Medicine, University of Ulsan, 88, Olympic-Ro 43-Gil, Songpa-Gu, Seoul, 05505 South Korea; 2 Department of Ophthalmology, Dongguk University Ilsan Hospital, Goyang, South Korea; 3 Department of Medical Science, Asan Medical Center, AMIST, University of Ulsan College of Medicine, Seoul, South Korea; 4 Translational Biomedical Research Group, Asan Institute for Life Science, Asan Medical Center, Seoul, South Korea; 5 Genome Insight Technology Inc, Daejeon, South Korea; 6 Department of Ophthalmology, Yeungnam University College of Medicine, Daegu, South Korea

**Keywords:** miRNA, Central serous chorioretinopathy, miR-184, Next-generation sequencing, Choroidal endothelial cell

## Abstract

**Background:**

Central serous chorioretinopathy (CSC) is the fourth most prevalent retinal disease leading to age-related macular degeneration (AMD) and retinal atrophy. However, CSC's pathogenesis and therapeutic target need to be better understood.

**Results:**

We investigated exosomal microRNA in the aqueous humor of CSC patients using next-generation sequencing (NGS) to identify potential biomarkers associated with CSC pathogenesis. Bioinformatic evaluations and NGS were performed on exosomal miRNAs obtained from AH samples of 62 eyes (42 CSC and 20 controls). For subgroup analysis, patients were divided into treatment responders (CSC-R, 17 eyes) and non-responders (CSC-NR, 25 eyes). To validate the functions of miRNA in CECs, primary cultured-human choroidal endothelial cells (hCEC) of the donor eyes were utilized for in vitro assays. NGS detected 376 miRNAs. Our results showed that patients with CSC had 12 significantly upregulated and 17 downregulated miRNAs compared to controls. miR-184 was significantly upregulated in CSC-R and CSC-NR patients compared to controls and higher in CSC-NR than CSC-R. In vitro assays using primary cultured-human choroidal endothelial cells (hCEC) demonstrated that miR-184 suppressed the proliferation and migration of hCECs. STC2 was identified as a strong candidate for the posttranscriptional down-regulated target gene of miR-184.

**Conclusion:**

Our findings suggest that exosomal miR-184 may serve as a biomarker reflecting the angiostatic capacity of CEC in patients with CSC.

**Supplementary Information:**

The online version contains supplementary material available at 10.1186/s12951-023-02019-6.

## Introduction

Central serous chorioretinopathy (CSC) is the fourth most prevalent retinal condition, affecting 10 men and two women per 100,000 annually [1, 2]. Major pathogenesis of CSC is choroidal hyperpermeability and fluid leaking into the subretinal space through the retinal pigment epithelium (RPE), resulting in substantial central vision impairment [3]. The condition is characterized by choroidal vascular pathology (e.g., choroidal thickening [pachychoroid], choroidal venous congestion), with choroidal endothelial cells (CECs) playing a key role in disease progression [4]. CECs are fenestrated and essential for controlling the trans-endothelial transport and pressure of the choroidal vessels in patients with CSC [5–7]. Most patients recover spontaneously within 3 months of onset; however, the condition can recur or persist for longer periods and require intervention [1, 4]. In these cases, active treatments such as photodynamic therapy, laser photocoagulation, mineralocorticoid receptor antagonist, and intravitreal anti-vascular endothelial growth factor (anti-VEGF) injection may be required [3, 4].

In patients with CSC, anti-VEGF therapy is used to reduce the hyperpermeability of choroidal endothelial cells [8]. Despite the lack of comprehensive randomized clinical trials, anti-VEGF agents such as bevacizumab, ranibizumab, and aflibercept have been reported to benefit patients with CSC by lowering the level of subretinal fluid [9–12]. Although CSC and AMD are different disease entities, both have several common characteristics, such as choroidal hyperpermeability and neovascularization [4, 8, 9]. Also, patients with CSC can progress into exudative AMD [13]. However, the pathogenesis and therapeutic target for CSC are poorly understood; therefore, we have limited information on patients who do not benefit from anti-VEGF treatment and have a suboptimal response, such as those with AMD [8, 14]. Discrimination of this subset of patients during evaluation may be practical when designing the treatment strategy.

Exosomes are nanovesicles (50–175 nm in diameter) composed of a lipid bilayer containing proteins and nucleic acids that can be utilized as biomarkers [15]. Exosomes are released into the extracellular environment by cells and are detected in various body fluids such as serum, urine, saliva, and aqueous humor (AH) [16–18]. Proteins and lipids are the major constituent of exosomes, and RNAs such as messenger RNA (mRNA) and small RNAs, which are transported to nearby target cells, are also involved [15]. Exosomes are emerging as key indicators of human disorders given their involvement in cell-to-cell communication, in part through exosomal RNA transport [19].

MicroRNAs (miRNAs) are single short-stranded (19–22nt) RNAs that regulate the posttranscriptional process by degradation or inhibition of the target [20]. Their role in gene regulation at development, proliferation, and differentiation is considered as essential as that of gene silencing mediators [21]. Recent reports suggest that miRNAs are found in AH and are expected to be important markers of ocular diseases [17, 18, 22, 23]. Exosomes in AH are considered the transporters of miRNAs in cell-to-cell communication in the eye. Therefore, profiling the exosomal miRNA in AH could offer an important clue to the pathophysiology of CSC. Herein, we aim to characterize the exosomal miRNA of patients with CSC and investigate its contribution to CSC pathogenesis and treatment response to anti-VEGF.

## Results

### Exosomal miRNA analysis by NGS in aqueous humor of patients with CSC

**Fig. 1:**
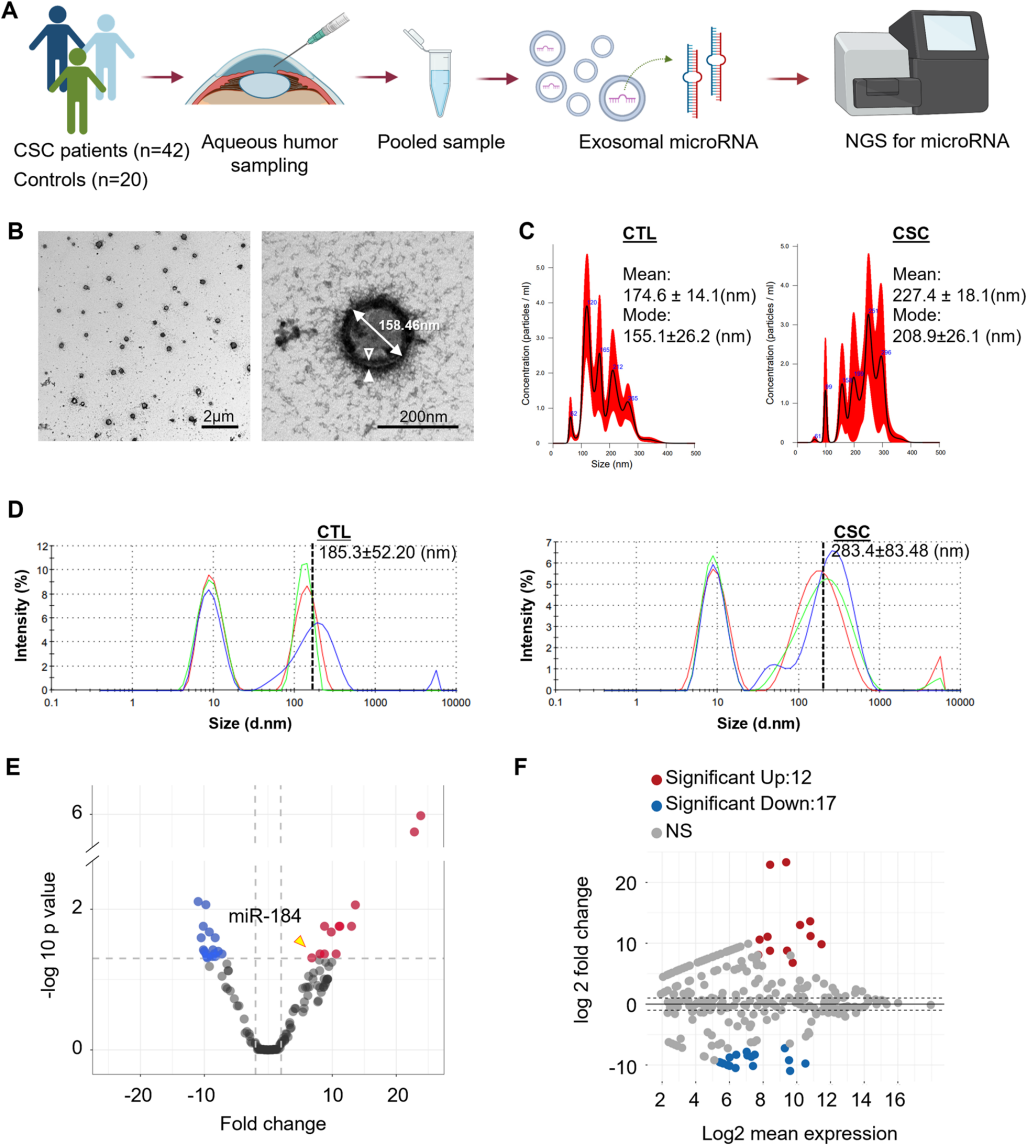
Exosomal miRNA expression profiles of aqueous humor (AH) derived from controls and patients with central serous chorioretinopathy (CSC). **A** Schematic depicting the experimental flow of the aqueous humor exosomal sequencing. **B** Transmission electron microscope image of exosome in aqueous humor from a single patient. The exosomes were concentrated to 10 × after ultracentrifugation. Purified exosomes have a double membrane (inner membrane; blank arrowhead, outer membrane; filled arrowhead), and the size of the exosome is indicated (double-headed arrow). **C** Nanoparticle tracking analysis (NTA) result of nano-size particles (exosomes) of aqueous humor from a single control and patient with CSC. Patients with CSC showed larger mean and mode vesicle sizes than the control (CTL). **D** Fresh frozen patient aqueous humor nanoparticle distribution in DLS measurement. The left graph shows controls (CTL, three patients), and the right graph shows CSC (three patients). The value represents the most intense nanoparticle size ± standard error. Note that intense nanoparticle (exosome) size distribution is larger in CSC than in the control. **E**, **F** Volcano plot **E**, and MA plot **F** indicate differentially expressed miRNAs between the control and CSC patients. NS for no statistical significance. Dotted line represents (**E**) log2 fold change > 2 and *P* < 0.05, and (**F**) log2 fold change > 2.

A total of 62 eyes (42 CSC and 20 control) were included in this study (Fig. 1A). Among the CSC eyes, 34 were men and eight were women (50.8 ± 8.6 years), while control eyes comprised five men and 15 women (62.2 ± 3.6 years). Analysis of baseline characteristics and baseline optical coherence tomography (OCT) parameters are described in Table 1. AH was collected and pooled (3.0 mL) for analysis (Fig. 1A). Before profiling exosomal miRNA expression, we purified and confirmed the exosomes in the AH of the patients using TEM, and analyzed the exosomes using NTA and DLS (Fig. 1B). The average size and concentration of the exosomes increased in patients with CSC compared with controls (Fig. 1C, D). In the exosomal miRNA profiling result, a total of 376 miRNAs were consistently detected by NGS across all groups. Compared with controls, patients with CSC had 12 significantly upregulated miRNAs and 17 down-regulated miRNAs (Fig. 1E, F). After analyzing differentially expressed miRNAs by fold change and volcano plot filtering, we confirmed that miR-184 was significantly upregulated in CSC compared with controls (Fig. 1E).

**Table 1:** Baseline Characteristics on the CSC and Control Eyes

	Control n = 20	CSC n = 42	p-value
Sex (male: female)	5: 15	34: 8	< 0.001^*^
Age (years)	62.2 ± 3.6	50.8 ± 8.6	< 0.001^†^
Baseline SFChT (µm)	233.92 ± 95.24	372.08 ± 104.60	< 0.001^†^

^*^Chi-square test, ^†^Unpaired t-test

*CSC* central serous chorioretinopathy, *SFChT* subfoveal choroidal thickness

### Subgroup analysis according to intravitreal anti-VEGF treatment response

**Fig. 2:**
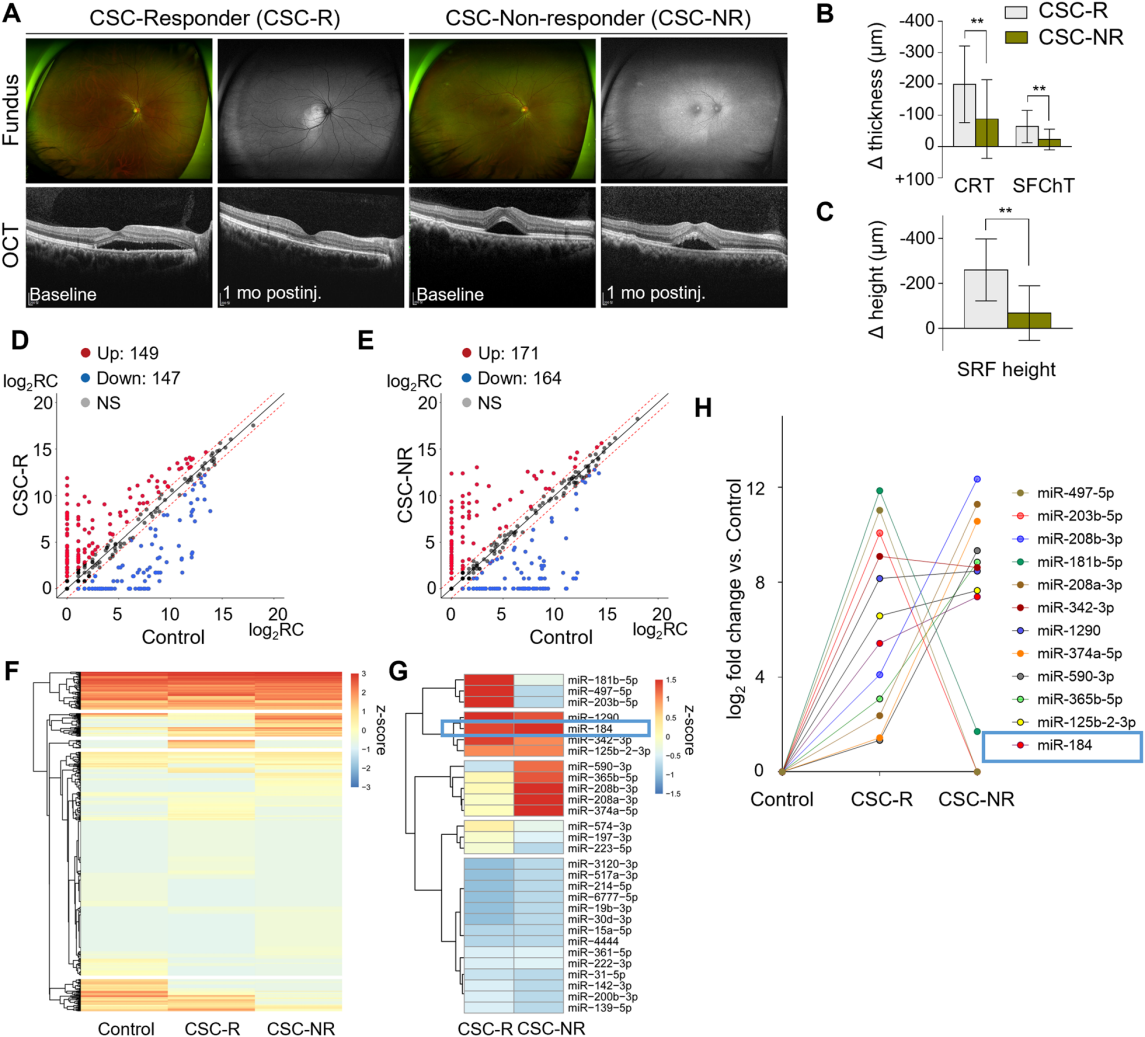
Subgroup analysis of the patients with CSC according to the treatment response to intravitreal anti-VEGFs. **A** Representative wide-field fundus, autofluorescence images, and optical coherence tomography (OCT) of patients with CSC depending on the anti-VEGF treatment response. **B**, **C** Quantification of the changes to the OCT parameters; central retinal thickness (CRT), subfoveal choroidal thickness (SFChT), and subretinal fluid (SRF) height changes were measured. (n = 17, responder [CSC-R]; n = 25, non-responder [CSC-NR]). **D**, **E** Scatter plots show the difference in the expression of miRNAs between control and CSC-R (**D**) and CSC-NR (**E**). The red-dotted line indicates twofold changes. NS for no statistical significance. **F** Heatmap of unsupervised hierarchical clustering. (**G**) Heatmap of differential miRNA expression between CSC-R and CSC-NR. (**H**) Relative miRNA expression in AH-derived exosomes from controls or patients with CSC-R or CSC-NR

Patients with CSC-R and CSC-NR were compared to gain insight into the miRNA expression differences between the anti-VEGF treatment response (Fig. 2). Among the 42 patients with CSC, 17 eyes were classified as CSC-R (treatment responder), and 25 were classified as CSC-NR (treatment non-responder) (Fig. 2A). The differences in baseline clinical characteristics and OCT findings between the two groups were not significant (Table 2). The mean values of CRT, SFChT, and SRF height in the CSC-Rs group decreased significantly in the CSC-NRs group at 1 month after IVB injection (Fig. 2B, C). Figure 2D–H shows exosomal miRNA expression patterns in patients with CSC according to treatment response. Differentially expressed miRNA results showed that miR-184 (hsa-miR-184) is an upregulated miRNA in patients with CSC-R and CSC-NR compared with controls (Fig. 2D–G). Moreover, miR-184 was significantly upregulated in patients with CSC-NR compared with CSC-R (Fig. 2F–H). Therefore, we focused on identifying the role of miR-184 in patients with CSC.

**Table 2:** Comparison of clinical characteristics and OCT findings between the CSC responders and CSC non-responders

	CSC Responders (n = 17, 40.5%)	CSC non-responders (n = 25, 59.5%)	*p*-value
Clinical characteristics			
Sex (male: female)	14: 3	20: 5	
Age (years)	51.1 ± 7.5	50.5 ± 9.4	0.965^†^
Hypertension (yes: no)	4: 13	3: 22	0.325^*^
Diabetes (yes: no)	0: 17	0: 25	1.000^*^
Visual acuity (LogMAR)	0.251 ± 0.317	0.211 ± 0.269	0.662^†^
Duration of symptom (wk)	10.5 ± 4.8	8.6 ± 3.9	0.162^†^
First event: Recurrence	14: 3	21:4	0.888^*^
Baseline OCT findings			
Baseline CRT (µm)	424.65 ± 129.56	458.52 ± 122.41	0.395^†^
Baseline SFChT (µm)	379.86 ± 118.08	366.82 ± 96.58	0.697^†^
Baseline SRF height (µm)	256.25 ± 164.81	225.94 ± 126.07	0.504^†^
Baseline PED height (µm)	63.18 ± 38.18	61.51 ± 96.04	0.938^†^
Changes of OCT findings at 1 month after bevacizumab			
Δ CRT (µm)	− 198.73 ± 122.32	− 88.00 ± 125.43	0.010^†^
Δ SFChT (µm)	− 63.92 ± 51.81	− 23.00 ± 33.24	0.005^†^
Δ SRF height (µm)	− 259.73 ± 138.00	− 67.18 ± 121.72	< 0.001^†^
Δ PED height (µm)	− 28.94 ± 41.48	− 11.69 ± 30.88	0.146^†^

^*^Chi-square test, ^†^Unpaired t-test

*CSC* central serous chorioretinopathy, *CRT* central retinal thickness, *LogMAR* Logarithm of the Minimum Angle of Resolution, *SFChT* subfoveal choroidal thickness, *SRF* subretinal fluid, *PED* pigment epithelial detachment

### Exosomal miR-184 expression in aqueous humor of individual patients with CSC

**Fig. 3:**
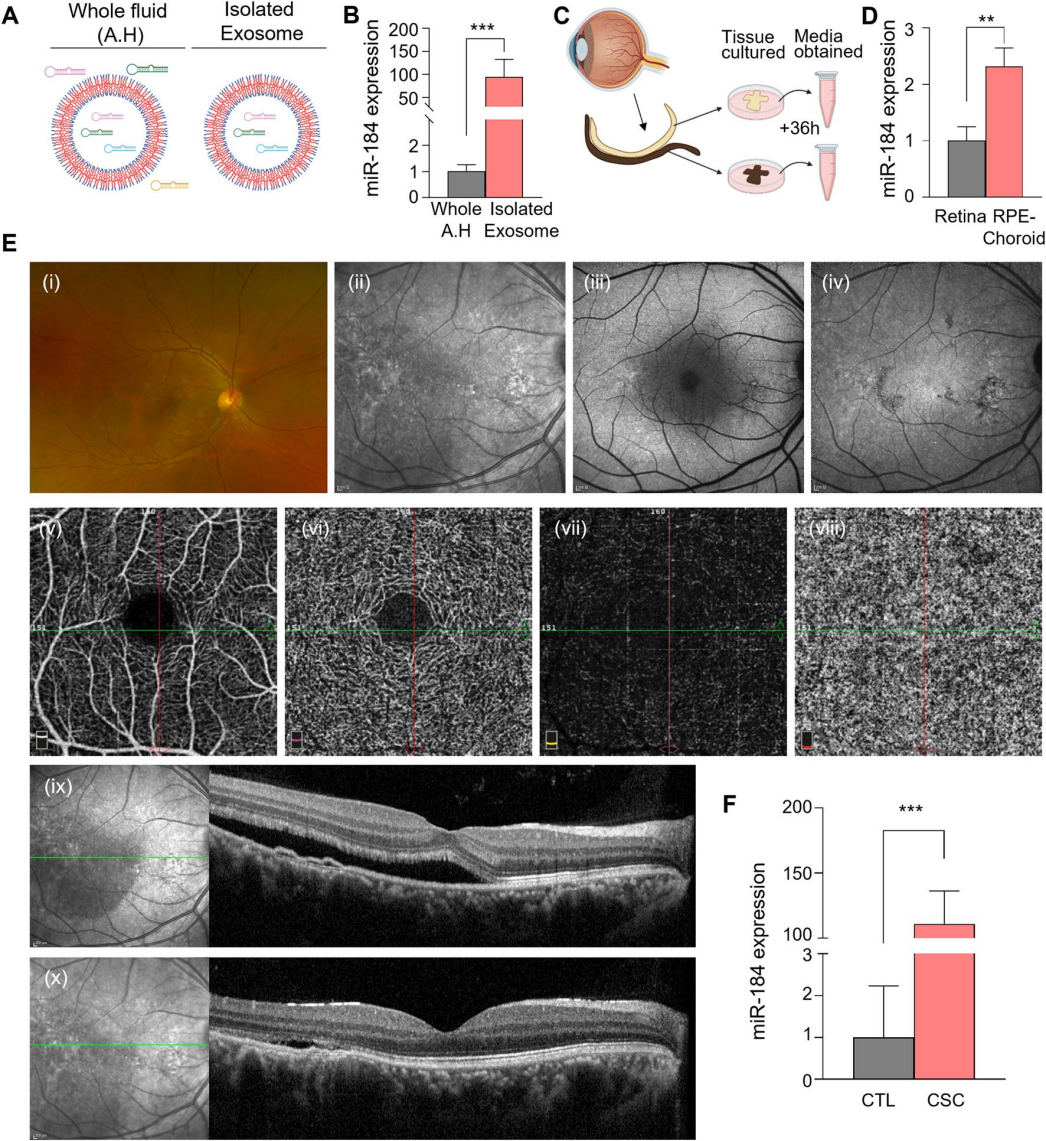
miR-184 is upregulated in aqueous humor-derived exosomes from patients with CSC. **A** Schematic of whole aqueous humor fluid (AH) and isolated exosome to depict location, distribution, and concentration of miRNAs of interest. **B** miR-184 detection using probe-based qPCR in aqueous humor fluid of the control and purified exosome from the same aqueous humor sample. Expression of miR-184 isolated from the purified exosomes was significantly higher, indicating that miR-184 is more concentrated in exosomes. **C** Schematic of tissue culture from a fresh donor eye and media obtained for analysis of the secreted exosomes from the retina and RPE-choroid of the fresh cultured tissues. **D** miR-184 detection using probe-based qPCR in the retina or RPE-choroid tissue cultured media. miR-184 was significantly higher in RPE-choroid cultured media in the first 36 h after fresh incubation. **E** Representative multimodal images of a patient with subacute CSC. (i) UWF (ii) SLO-IR (iii) BAF (iv) IRAF (v) OCTA (SCP) (vi) OCTA (DCP) (vii) OCTA (OPL-BRM) (viii) OCTA (choriocapillaris) (ix) OCT at baseline (x) OCT after 1-month treatment of anti-VEGF therapy. **F** Exosomal miR-184 expression from the aqueous humor of the patient with CSC (**E**) is measured and relatively quantified compared with the control. Four independently synthesized miRNA cDNAs were amplified with two replicates per cDNA to reduce the technical error. Note the significant elevation of miR-184 expression. The graph values are represented as mean ± standard deviation. Statistical significance indicated as *P < 0.05, **P < 0.01, ***P < 0.001

To validate exosomal miR-184 expression in the AH of patients with CSC, we isolated exosomes from AH and compared miR-184 expression with that of the whole AH fluid (Fig. 3A). Notably, miR-184 expression was significantly upregulated (near 100-fold increase) in isolated exosomes compared with whole AH, demonstrating the presence of concentrated miR-184 s inside the exosomes rather than AH fluid (Fig. 3B). To investigate the origin of exosomal miR-184 in the eye, we performed a tissue culture of the retina and the RPE-choroid complex in a donor eye (Fig. 3C). After 36 h of tissue culture, exosomal miR-184 expression was above twofold higher in the exosomes in the media of the RPE-choroid compared with those in retinal media, indicating that exosomal miR-184 primarily originates from the RPE-choroid rather than the retina (Fig. 3D). To validate the expression of AH exosomal miR-184 in individual patients, we performed qPCR analysis of miR-184 in CSC and control AH exosomes. A subacute CSC patient with decreased visual acuity for 2 months was selected based on multimodal imaging (Fig. 3E). Comparable with the miRNA-sequencing results, the patient had an increased level of miR-184 expression (about 100-fold) compared with a control, confirming miR-184 elevation in patients with CSC who had a suboptimal response to the anti-VEGF treatment (Fig. 3F).

### Functional and disease association analysis for differentially expressed miRNAs of patients with CSC

**Fig. 4:**
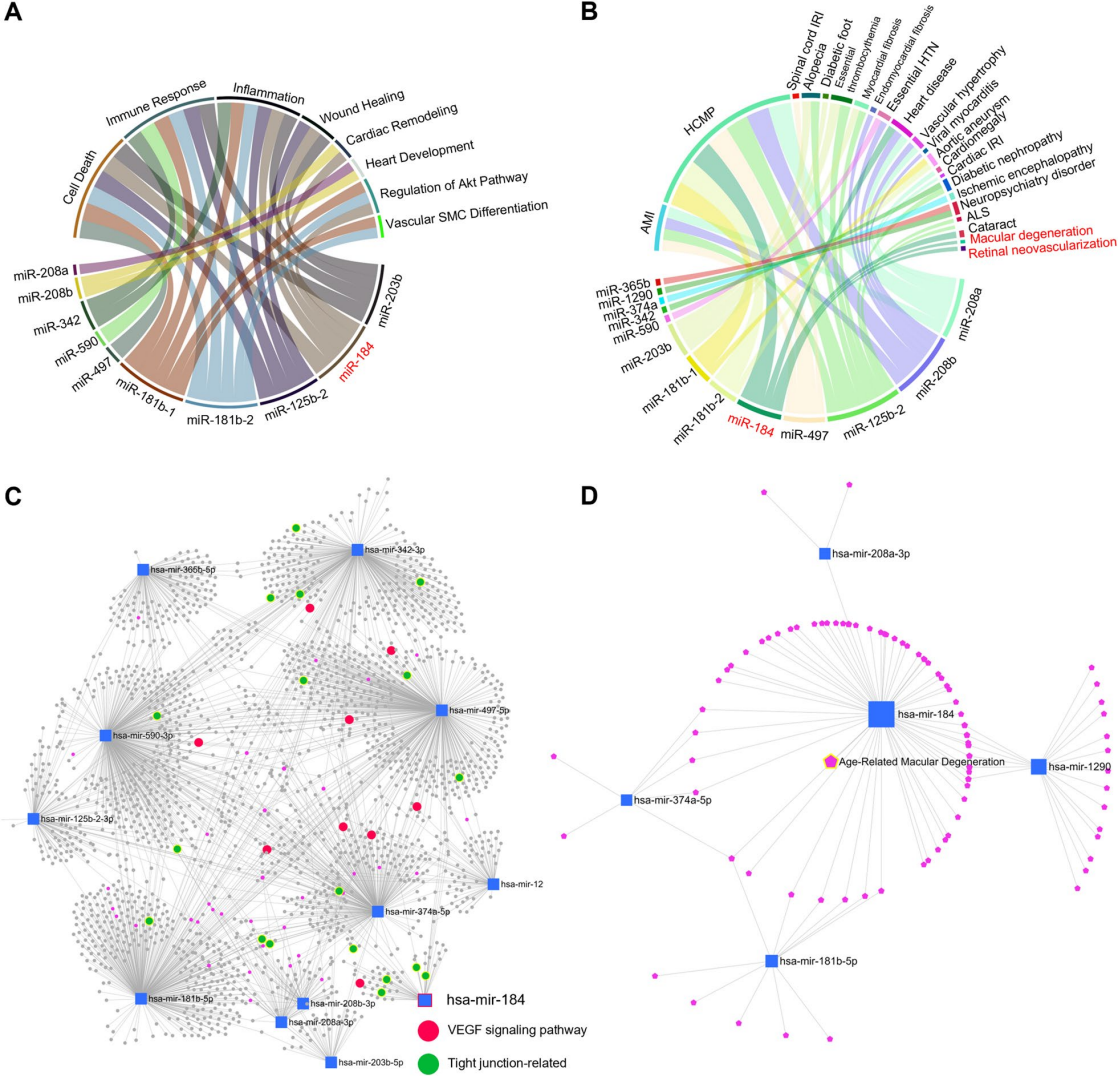
Functional and disease relationship of differentially expressed miRNAs of patients with CSC.** A** Chord diagram showing the differentially expressed miRNAs connection between enriched biological processes (by colors). **B** Chord diagram showing the differentially expressed miRNAs and linking enriched disease association (by colors). **C** Graphical representation of the posttranscriptional network of interactions between the upregulated miRNAs and downregulated target genes. Gene ontology clusters of the VEGF signaling pathway and tight junction-related pathway are colored. **D** Network analysis showing upregulated miRNA-related diseases. Age-related macular degeneration is highlighted

Gene ontology of the biological processes was analyzed to examine the functional effects of miRNAs (Fig. 4A). All upregulated miRNAs involved in functional pathways were identified using an FDR cutoff value of 0.05 [24]. We noted that miR-184 was significantly associated with cell death, inflammation, wound healing, cardiac remodeling, and regulation of the Akt pathway. To explore the physiological impact of the differentially expressed miRNAs, miRNAs possibly associated with cardiovascular and CNS-related disorders were also assessed (Fig. 4B). Notably, we identified retinal dysfunctions such as macular degeneration and retinal angiogenesis related to miR-184 (Fig. 4B and D). In addition, gene network analysis revealed that upregulated miRNAs in patients with CSC, including miR-184, were significantly related to VEGF signaling and tight junction pathways, which are closely related to the regulation of vascular permeability (Fig. 4C) [25–28]. Therefore, we focused our analysis on miR-184, which could be considered a contributing factor to the pathophysiology and development of the suboptimal response to anti-VEGF in CSC, or as a compensatory factor is elevated to prevent the development of CSC.

### miR-184 suppresses angiogenesis and migration of the choroidal endothelial cells

**Fig. 5:**
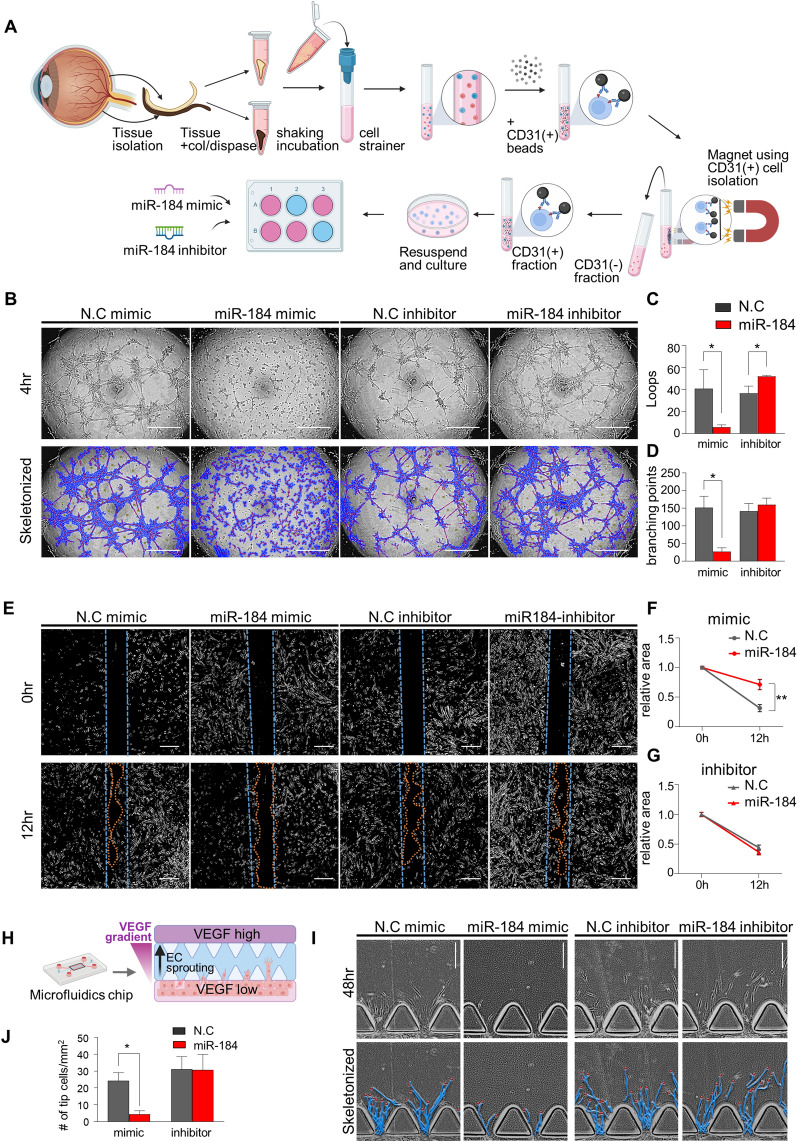
miR-184 suppresses growth and migration of human primary choroidal endothelial cells (hCEC) **A** Schematic of isolation of endothelial cells from fresh donor eyes using CD31( +) magnetic beads and culture and assay of miR-184 mimic and inhibitor transfected cells. **B** 3D Tube formation assay using miR-184 mimic/inhibitor transfected hCEC (primary human choroidal endothelial cell) at 4 h after seeding. Images were skeletonized and measured for analysis. Scrambled negative controls for mimic and inhibitor are indicated as N.C. **C**, **D** Total **C** loops and **D** branching points were measured for tube formation property parameters at 4 h. Note a significant decrement of total loops and branching points at miR-184 mimic transfected cells. **E** Wound healing assay using miR-184 mimic/inhibitor-transfected hCEC. The wound space was measured at 2 h after scratching. **F**, **G** Measurement of the wound space area. **F** miR-184 mimic transfected cells showed reduced wound closure, while **G** no significant difference was observed in the miR-184 inhibitor transfected cells. **H** Schematic of sprouting angiogenesis assay using microfluidics chip. 20 ng/mL of VEGF gradient was generated to induce endothelial cell sprouting. **I** Sprouting angiogenesis assay using miR-184 mimic/inhibitor transfected hCEC. Cells were analyzed at 48 h after generating a VEGF gradient. **J** The number of tip cells per mm was measured and analyzed. miR-184 mimic transfected cells express significantly low tip cells. All assays were performed with three replicative cultures. Graph values are represented as mean ± standard deviation. Statistical significance indicated as *P < 0.05, **P < 0.01, (all n = 3), **B**, **C** Scale bar = 1 mm. **E**–**I** Scale bar = 20 μm

To elucidate the meaning of the elevation of miR-184 in patients with CSC, we validated the functional role of miR-184 in choroidal endothelial cells by performing in vitro assays using miR-184 transfection in primary cultured-human choroidal endothelial cells (hCEC) (Fig. 5A). Before the examination, we confirmed in a previous report that miR-184 targets the nervous system [29]. Moreover, previous research has indicated that miR-184 negatively regulates angiogenesis in corneal epithelial cells and human umbilical vein endothelial cells, suggesting a possible role in regulating the angiogenic properties of hCEC [30, 31]. Interestingly, miR-184 mimics significantly decreased tube formation of the hCEC, evidenced by a decreased number of endothelial cell loops and branching points, while the miR-184 inhibitor significantly increased total loops (Fig. 5B–D). Moreover, a wound healing assay of the hCEC revealed that miR-184 mimics significantly suppressed endothelial cell migration, whereas miR-184 inhibitor had no significant effect on migration (Fig. 5E–G). We conducted a microfluidics chip assay to evaluate the effect of miR-184 on sprouting angiogenesis as a response to VEGF (Fig. 5H). miR-184 mimics significantly decreased endothelial sprouting, evidenced by a decreased number of endothelial tip cells 48 h after exposure to the VEGF gradient (Fig. 5I, J). Overall, these in vitro results suggest that miR-184 suppresses the angiogenesis of hCEC, thereby indicating that its elevation can be translated into a compensatory mechanism to prevent angiogenic properties of CEC in patients with CSC.

### STC2 is a direct target gene of miR-184

**Fig. 6:**
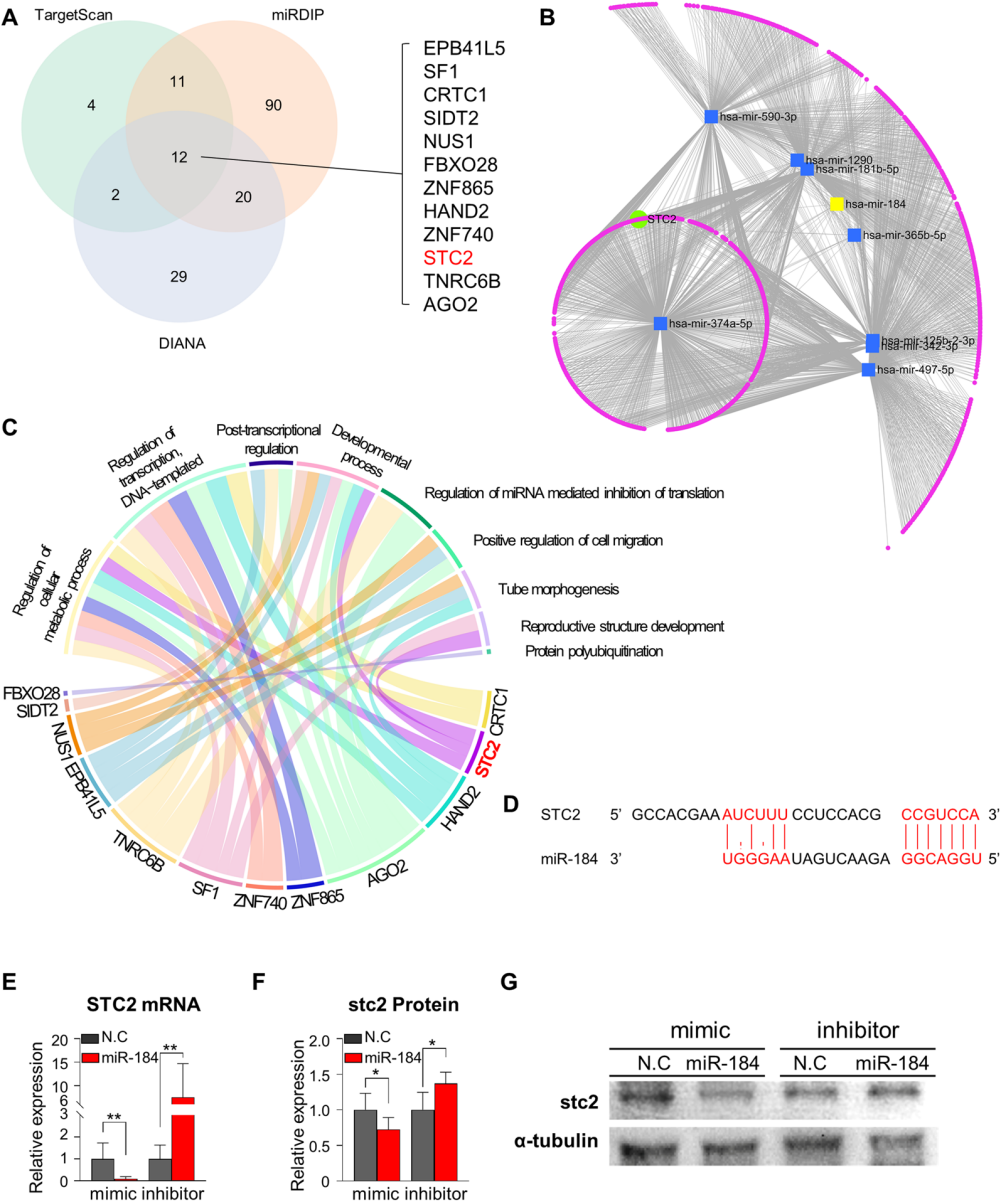
STC2 is a potential target for miR-184 regulating angiogenesis, vasculogenesis, motility, and choroidal endothelial cell proliferation. **A** Venn diagram showing miR-184 target gene prediction results of TargetScan, miRDIP, and DIANA database. **B** Target gene interaction network for miRNAs enriched in the exosome of AH in patients with CSC. *STC2* and miR-184 are highlighted. **C** Chord diagram showing the target genes of miR-184 and linking between enriched biological processes (by colors). **D** Prediction of miR-184 binding sites in the 3’-UTR of human *STC2* mRNA. **E** The *STC2* mRNA was quantified in miR-184 mimic/inhibitor transfected hCEC 24 h after transfection. Four independent culture replicates with two amplification replicates were performed. **F-G** The STC2 protein in miR-184 mimic/inhibitor transfected was quantified by western blotting 48 h after transfection**.** Three independent culture replicates with two SDS-page replicates were performed. The graph values are represented as mean ± standard deviation. ^*^ *P* < 0.05, ^**^ *P* < 0.01 (all n = 3)

**Fig. 7:**
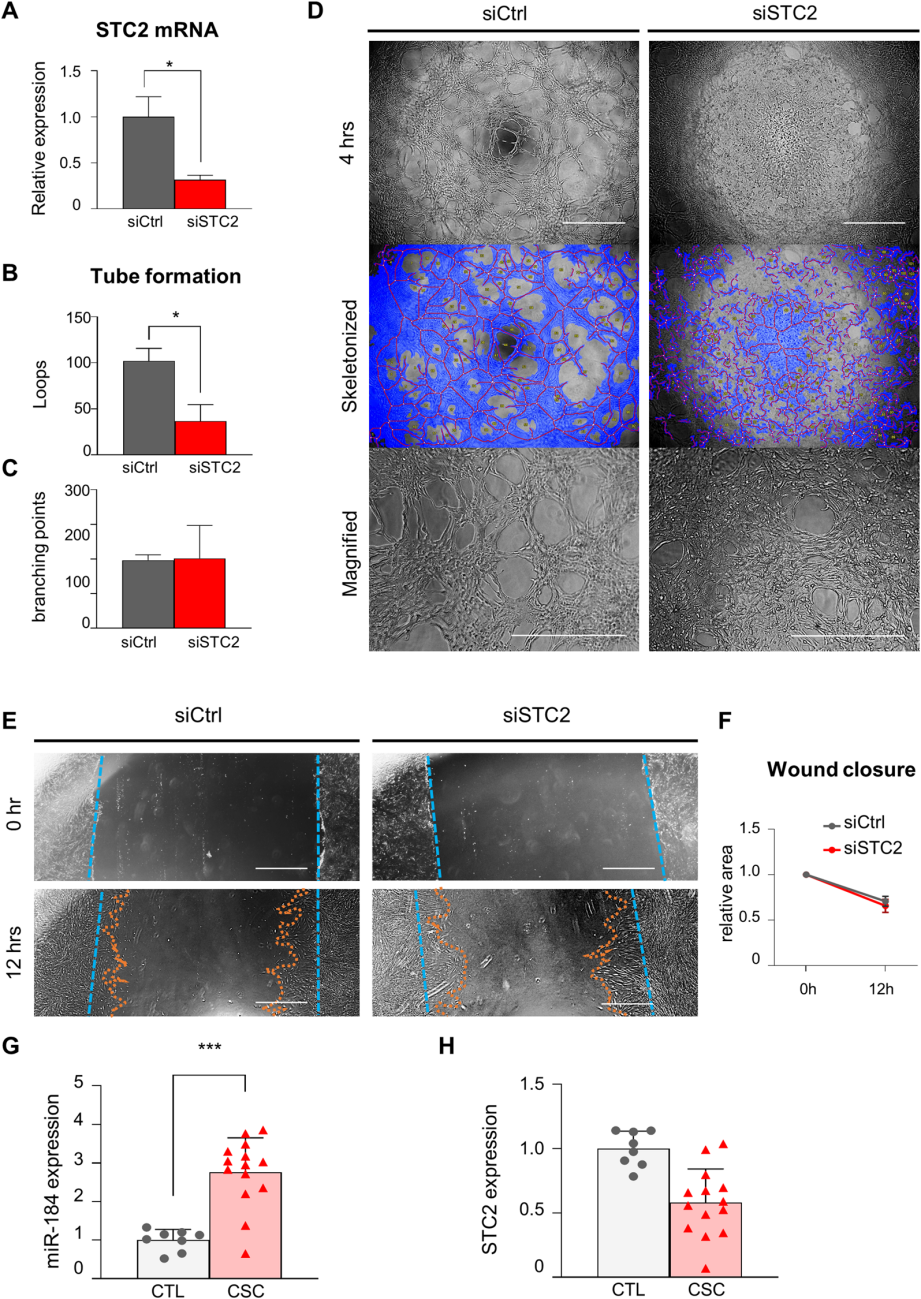
Downregulation of STC2 promotes angiogenesis in choroidal endothelial cells. **A** The STC2 mRNA was quantified in STC2 siRNA transfected (STC2 knock-down) hCECs 24 h after transfection. **B-C** siSTC2 treated cells were seeded for tube formation at 24 h after transfection. Total loops and branching points were measured for tube formation property parameter at 4 h. Note that significant decrement of total loops and branching points at STC2 knock-down cells. (n = 3 individual assays) **D** 3D Tube formation assay using STC2 knock-down hCECs at 4 h after seeding. Images were skeletonized, magnified and measured for analysis. **E** Scratch wound closure assay using siSTC2-treated hCECs. The wound space was measured at 12 h after scratching. **F** The wound space area was measured (n = 3 individual assays). **G-I** Individual patient-derived exosomal miR-184 quantification **G** and *STC2* mRNA quantification **H** of controls and patients with CSC (n = 8 for controls, n = 14 for CSC)

To gain insight into the targets of miR-184 in CSC pathophysiology, we aimed to predict target genes by Venn diagram analysis (Fig. 6A). Twelve common target genes were identified in the TargetScan, miRDIP, and DIANA databases. We focused on *STC2,* given it was the most highly suppressed angiogenesis-related gene other than transcriptional factors by miR-184 mimic in hCEC (Additional file 1: Fig. S1) [32]. Furthermore, network analysis of upregulated miRNAs validated the strong linkage of *STC2* and miR-184 (Fig. 6B), which rationalized the evaluation of *STC2* as a strong candidate for miR-184 target genes. To predict the functional effects of common target genes of miR-184, including *STC2*, we analyzed the gene ontology of biological processes (Fig. 6C). We observed that *STC2* was significantly related to cell development and metabolic regulation, and through bioinformatic prediction, we verified miR-184 binding sites in the 3’-UTR of human *STC2* mRNA (Fig. 6D). To determine whether miR-184 influences *STC2* expression transcriptionally, we measured *STC2* mRNA and protein levels in hCEC after transfection and inhibition experiments with miR-184 mimic and inhibitor. We observed that miR-184 mimic significantly downregulated and miR-184 inhibitor significantly upregulated *STC2* mRNA levels (Fig. 6E, F). The protein levels of *STC2* followed the same trend (Fig. 6G), confirming that miR-184 suppresses *STC2* expression in hCEC. To gain insight into the role of STC2 in hCECs, we performed in vitro tube formation and wound healing assay using siRNA of *STC2*. Knock-down of *STC2* remarkably reduced tube formation of hCEC, but the wound closure did not show a significant difference to the controls (Fig. 7A–F). This result suggests the possibility that STC2 may be a downstream target of miR-184, which has a more role in angiogenesis rather than cell migration. We further elaborated qPCR analysis of *STC2* and miR-184 in exosome of the patients who were eligible for simultaneous analysis of miR-184 and STC2 mRNA (Additional file 2). Interestingly, CSC patients showed significantly higher expression of exosomal miR-184 and a trend of lower *STC2* mRNA expression than the controls (Fig. 7G–H). Collectively, these results serve as a rationale for miR-184 potentially being a superior biomarker compared to STC2.

**Fig. 8:**
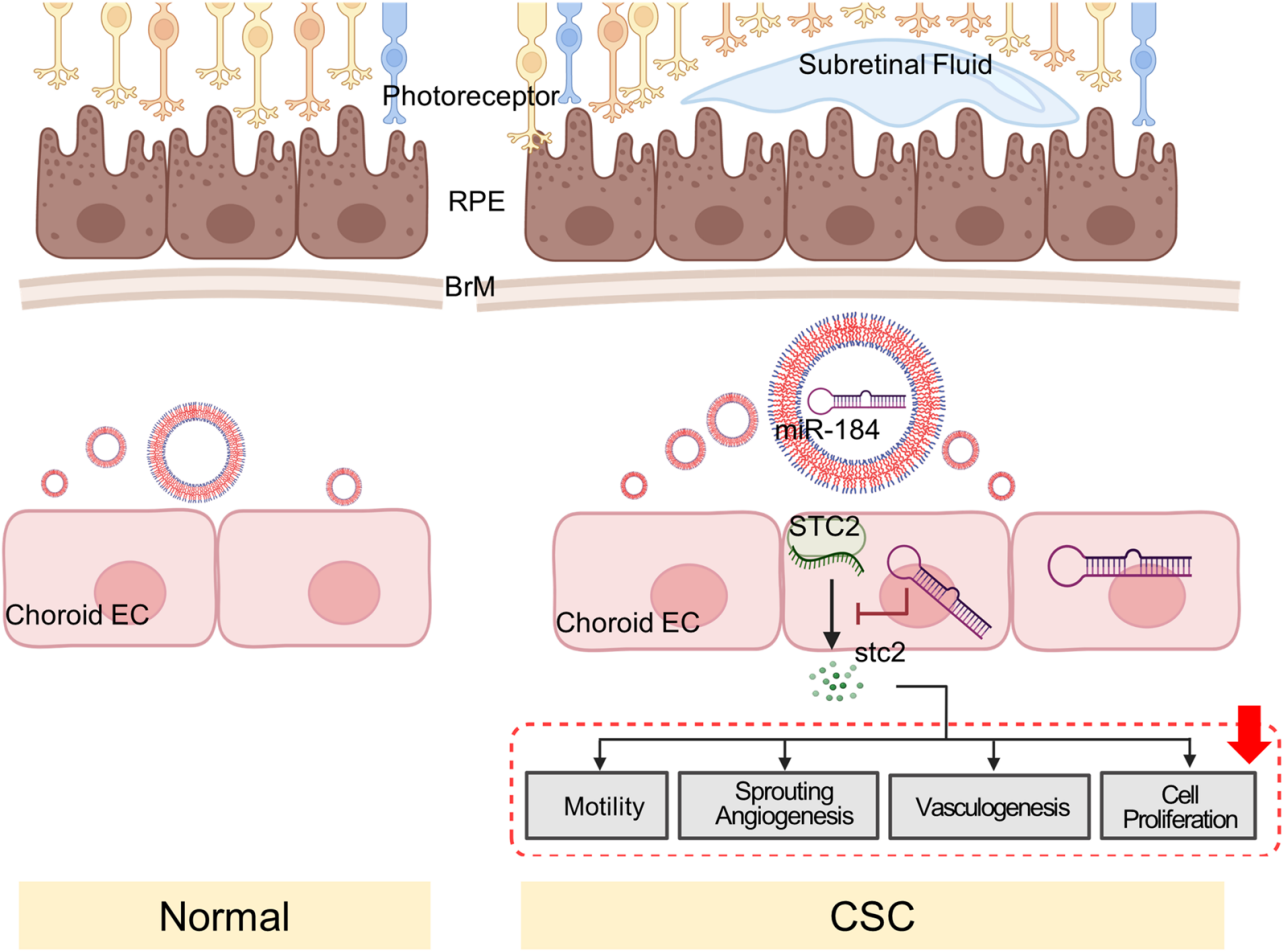
Schematic of the summary of exosomal miR-184 function in CSC choroidal endothelial cells. Exosomes produced in CSC choroid EC containing miR-184 are delivered to neighboring EC to inhibit the angiogenic pathway by inhibiting *STC2* transcription in Choroid EC, and results in reduced STC2 protein production. Thus, the angiogenic properties were inhibited and indicated in the red dashed box with a solid red arrow. Schematic figures were created with BioRender

Overall, our results suggest that miR-184 may impact several cellular processes involved in angiogenesis, vasculogenesis, EC motility, and cellular proliferation, likely by targeting *STC2* in CEC (Fig. 8). miR-184 may be compensatively elevated in patients with CSC with high angiogenic properties of CEC. Therefore, miR-184 could serve as a potent biomarker for identifying patients with high angiogenic-CSC and predicting their suboptimal anti-VEGF treatment response.

## Discussion

By analyzing AH-derived exosomes in patients with CSC, we report for the first time that exosomal miR-184 is enriched in patients with CSC compared with controls, and significantly affects hCEC functions such as angiogenesis. miR-184 prevents angiogenesis in hCEC and can be elevated to prevent angiogenic properties in patients with CSC. We suggest that miR-184 targets *STC2* to suppress import cellular processes of CEC, such as choroidal neovascularization, hyperpermeability, and retinal degeneration. Therefore, miR-184 reflects the angiostatic properties of CEC and can be used as a promising biomarker for predicting anti-VEGF treatment response.

Since acquiring a retinal biopsy specimen is challenging and bears the considerable danger of neural damage that can threaten vision, AH is considered an excellent surrogate for retinal tissue. Recent research has demonstrated that miRNAs in human AH exist in both solutions and are contained within exosomes, which provides important details on cell-to-cell communication in the eye [15, 17, 18]. Therefore, many studies have utilized AH to analyze miRNA to study the pathology of eye diseases such as AMD, glaucoma, and myopia [15–18, 33] Furthermore, miRNA in AH is eye-specific and plays a significant role in the development and pathology of the eye [22]. In this regard, our method that acquires information on AH miRNAs with safe, office-based, microscope-guided anterior chamber puncture can be an excellent tool to improve the understanding of retinal diseases.

Previously, photodynamic therapy (PDT) has been considered the standard treatment for such cases [4]. However, due to concerns of macular atrophy or secondary choroidal neovascularization (CNV) development after PDT, and the potential for superimposed polypoidal neovasculopathy or secondary CNV in CSC patients, preference of anti-VEGF therapy as an initial treatment before PDT is increasing [34, 35]. Therefore, in the case of subacute patients exhibiting symptoms for more than 6 weeks (it is possible that the actual disease duration is longer) as well as no definite improvement in macular anatomy, we consider intravitreal anti-VEGF injections, unless there are specific contraindications for the treatment. If the response is inadequate, we consider PDT as a secondary option. Our findings indicate that in over 40% of patients with CSC, a single injection of anti-VEGF resulted in complete fluid absorption, suggesting that anti-VEGF could be considered the primary treatment. Our study holds particular significance as research on biomarkers for assessing the treatment responsiveness of anti-VEGF therapy in patients with CSC has been limited.

To elucidate biomarker involvement in the treatment response of anti-VEGF with CSC, several reports show cytokines in aqueous humor. Lim et al. [36] reported no significant differences in VEGF and interleukin (IL)-8 levels of aqueous humor between patients with CSC and the control group. In other studies, however, the roles of VEGF and IL-8 in patients with CSC differ. For example, Jung et al. [37] observed higher VEGF levels in the aqueous humor of CSC-Rs than of CSC-NRs, while Terao et al. [38] showed IL-6 and IL-8 were more significantly upregulated in chronic CSC than in acute CSC. Many cytokine studies of patients with CSC have been conducted, but the findings are inconsistent. Therefore, we targeted the miRNA in exosomes released from the aqueous humor of the patients with CSC using NGS.

Notably, our data showed exosomal miR-184 was significantly enriched in CSCs (CSC-R and CSC-NR) compared with controls. Previous studies have demonstrated that miR-184 is fundamentally involved in neurological development and apoptosis and is an important regulator of oligodendrocytes, a potential candidate for neuroprotection [39–41]. Moreover, miR-184 is preferentially expressed in the eye, brain, and testis, and its expression was confirmed in the human cornea and the retina [42–45]. Comparable to the previous study, our exosomal miR-184 was abundant in AH [17]. It was also significantly enriched in the exosomes in the media of the human RPE-choroid tissue-cultured plate. Previous reports have demonstrated that miR-184 is involved in the ischemia and neovascular processes and significantly reduced in the retina [42]. miR-184 was suggested to be constitutively expressed to prevent the proangiogenic activity of the retina, and its downregulation indicates a complementary reaction for ischemia. In contrast to the study by Shen et al. [42], miR-184 was upregulated in CSC compared with controls and CSC-NR compared with CSC-R. We suggest that this miR-184 upregulation reflects increased baseline angiogenic activity, which might translate into increased vascular permeability in CSC. The degree of this upregulation is prominent in CSC-NR, which has higher choroidal vascular permeability and a lower response to intravitreal anti-VEGF compared with CSC-R. In this regard, analyzing the expression of miR-184 in AH can be an effective diagnostic method to measure the angiogenic activity of the choroid in CSC. Additionally, since the angiogenic activity is closely related to anti-VEGF response, higher angiogenic activity reflected as high levels of miR-184 in AH can serve as a surrogate biomarker for predicting response to anti-VEGF treatment in patients with CSC [46, 47]. Our study is limited by a lack of direct evidence regarding the role of miR-184 in regulating vascular permeability. Future longitudinal studies evaluating the relationship between AH miR-184 levels and the stability of vascular permeability or the development of choroidal neovascularization in a large population of CSC patients will clarify this issue.

We also demonstrated that miR-184 targets *STC2*, which is necessary for the angiogenesis and vasculogenesis of hCEC. *STC1* and *STC2* promote the angiogenesis of the EC through the involvement of VEGF/VEGFR2 and angiopoietin pathways [32]. *STC2* is expressed in the tumor vasculature and, when elevated, is positively associated with vascular invasion of the tumor [48, 49]. Ail et al. [50] reported that *STC2* gene expression is upregulated in the inner retina in hypoxic conditions. Although the function of *STC2* in the retina is understudied, we speculate its elevation to promote angiogenesis in the retina necessitates miR-184 increases to restrict *STC2* overexpression and maintain homeostasis between proangiogenic and anti-angiogenic factors in the retina. Future studies will confirm if *STC2* is responsible for proangiogenic activity and vascular hyper-permeability in patients with CSC.

Our study has some limitations such as small sample size and lack of information regarding the correlation between the expression level of miR-184 with retinal pathologies such as SFChT/CRT/SRF/PED in individual patients. However, achieving this would require a larger sample size for analysis, necessitating future prospective studies for more convincing results. Therefore, a large-scale longitudinal clinical study incorporating adjustments for various confounders will be necessary in the future. Nevertheless, even in the absence of statistically significant results, the result of our study which showed potential to quantify miR-184 level in individual patient samples is of considerable significance in understanding the expression levels and treatment responsiveness of individual patients.

In conclusion, our study delineates the potential role of exosomal miR-184 in AH as a biomarker reflecting choroidal endothelial status. Our research provides clues regarding the pathogenesis of the CSC and its treatment response after intravitreal anti-VEGF.

## Material and methods

### Patients

This prospective study included 42 eyes from 42 patients diagnosed with CSC in the Department of Ophthalmology at Yeungnam University College of Medicine, Daegu, Korea. Twenty eyes from 20 patients undergoing cataract surgery, without diabetes or diagnosed retinal disease, were selected as controls. For the exosome size analysis and miR-184 measurement of individual eyes, 15 patients with CSC and 22 with cataracts as controls were enrolled at Asan Medical Center. The study protocol was approved by the Institutional Review Board (IRB No. 2019-10-056-002 in Yeungnam University, IRB No. 2020-1945-0002 in Asan Medical Center). The study was performed in accordance with the tenets of the Declaration of Helsinki. The diagnosis of CSC was established by the presence of a typical fluorescein leakage pattern on fluorescein angiography (FA) and subretinal fluid accumulation evident on spectral domain optical coherence tomography (SD-OCT) [4]. We enrolled subacute CSC patients with a symptom duration ranging between 6 weeks and 4 months under the same criteria as a previous study [12]. Patients were excluded based on the presence of choroidal neovascularization, prior treatment for CSC, concurrent ophthalmologic disease or history of diabetes, any intraocular surgery, using systemic or topical carbonic anhydrase inhibitor within 1 month, and any history of intravitreal steroid injection to study eye.

### Multimodal imaging analysis of patients

All patients received bilateral ophthalmic examination, including biomicroscopic examination, fundus examination, and imaged with ultra-wide-field fundus photography (UWF, Optos California; Optos plc, UK), ultra-wide-field autofluorescence (UWF-AF) images, and ultra-wide-field FA (UWF-FA), or imaged with scanning laser ophthalmoscope infrared images (SLO-IR), blue autofluorescence (BAF), and infrared autofluorescence (IRAF), and the corresponding SD-OCT (Spectralis; Heidelberg Engineering, Heidelberg, Germany) [51]. Choroidal images were also obtained using the enhanced depth imaging (EDI) technique in SD-OCT. Ultra-wide-field indocyanine green angiography (UWF-ICGA) or optical coherence tomography angiography (OCTA) were performed as needed to rule out choroidal neovascularization. Central retinal thickness (CRT) was measured automatically on the central 1 mm zone from the fovea using SD-OCT segmentation analysis. Subfoveal choroidal thickness (SFChT) was manually measured by determining the vertical distance between the interface of the Bruch membrane and the sclerochoroidal junction on the B-scan of the OCT [52]. The height of the PED was assessed by measuring the vertical distance between the Bruch membrane and the apex of the retinal pigment epithelium (RPE) using the OCT scan in areas with the most prominent lesion [53]. SRF height was defined as the maximum distance between the RPE and the border of the detached neurosensory retina within a 3 mm nasal and 3 mm temporal area from the center of the fovea as previously described [54]. Measurement of SFChT, SRF height, and PED height were manually performed by utilizing a virtual caliper within the software (Heidelberg Eye Explorer ver. 1.10.2.0) [55] (Fig. 1A).

### Patient subgrouping

All patients with CSC were treated with intravitreal bevacizumab (IVB) and classified into responder and non-responder groups according to the result of the follow-up examination. Among the various anti-VEGF agents, some have not received approval for use in CSC, while IVB has been permitted for off-label use with IRB approval in our institute. Every patient was treatment naïve. CSC responders (CSC-Rs) were defined as patients who achieved complete absorption of SRF on SD-OCT at 1 month after IVB injection. CSC non-responders (CSC-NRs) were patients who remained SRF on SD-OCT at 1 month after IVB injection. Complete absorption was defined as the absence of SRF in an OCT 6 X 6 mm macular cube scan (Heidelberg Spectralis, Heidelberg, Germany); otherwise, it is defined as remaining SRF.

### Aqueous humor sampling

Aqueous humor (AH) samples were collected before IVB injection in the subacute CSC and during cataract surgery in the controls. To mitigate the occurrence of IOP spikes following injections [56], we performed a paracentesis procedure while sampling aqueous humor for study purpose prior to intravitreal injection after the permission from the patients. All the paracentesis procedure that we performed was very safe and prevented complications such as IOP surge. Patients were informed and signed a written consent form for the collection and scientific use of the specimen before the analysis. Approximately 100 to 200 µl of aqueous humor were collected from each patient, based on the amount of a previous study [16, 22]. The collected aqueous humor samples from the patients with CSC were sorted by the response to the bevacizumab as CSC responders or non-responders. Each group was pooled into Protein LoBind Tube. Pooling volumes of each group were control (N = 20) 3.5 mL, CSC responder (N = 17) 2.0 mL, and CSC non-responder (N = 25) 3.0 mL, and the mean volume for each group is 175 µl (3500/20), 117 µl (2000/17), and 120 µl (3000/25) for control, CSC responder, and CSC non-responder, respectively.

### Exosomal miRNA-sequencing

Exosomal RNA was isolated using ExoLutE^®^ Exosome Isolation Kits (Rosetta, Seoul, Korea), following the manufacturer’s instructions. Quality control and quantity measurement of the RNA was performed by Agilent 2100 Bioanalyzer using the RNA 6000 Pico Chip (Agilent Technologies, Amstelveen, The Netherlands). RNA quantification was performed using a NanoDrop 2000 Spectrophotometer system (Thermo Fisher Scientific, Waltham, MA, USA).

For control and test RNAs, library construction was performed using the NEBNext Multiplex Small RNA Library Prep kit (New England BioLabs, Inc., USA) according to the manufacturer’s instructions. Briefly, for library construction, 1 µg of each total RNA sample was used to ligate the adaptors, and then cDNA was synthesized using reverse-transcriptase with adaptor-specific primers. PCR was performed for library amplification, and libraries conducted clean-up using QIAquick PCR Purification Kit (Qiagen, Inc, German) and AMPure XP beads (Beckman Coulter, Inc., USA). The yield and size distribution of the small RNA libraries were assessed by the Agilent 2100 Bioanalyzer instrument for the high-sensitivity DNA assay (Agilent Technologies, Inc., USA). High-throughput sequences were produced by the NextSeq500 system as a way of single-end 75 sequencings (Illumina, SanDiego, CA, USA).

Raw and processed data have been deposited at GEO (https://www.ncbi.nlm.nih.gov/geo/) (accession no. GSE227142).

### Data analysis

Sequence reads were mapped by the bowtie2 software tool to obtain the bam file (alignment file). A mature miRNA sequence is used as a reference for mapping. Read counts mapped on mature miRNA sequences were extracted from the alignment file using bedtools (v2.25.0) and Bioconductor, which uses R (version 3.2.2) statistical programming language (R Development Core Team, 2011). Read counts were used to determine the expression level of miRNAs. The quantile normalization method was used for comparison between samples. Differentially expressed genes (DEGs) expression analysis was performed using DESeq2 [26]. Significant DEGs were defined as *P* < 0.05 and an absolute log2 fold change of > 2. For miRNA target and functional study, miRNet and miRWalk 2.0 were utilized [57, 58]. Graphical representations were generated using ggplot2.

### Single patient-derived aqueous humor preparation

Aqueous humor samples were delivered in individually sealed sterile syringes on ice, fresh or fresh-frozen. All samples were delivered to the lab and prepared within 4 h of acquisition. Fresh-frozen aqueous humor samples were thawed at 4 °C for 2 h. Fresh or fresh-frozen aqueous humor samples were filtered using a 4 mm RC membrane syringe filter (Corning, #431212, US) to minimize sample loss and collected at Protein LoBind Tube (Eppendorf, #022431081, Germany). The filtered aqueous humor was centrifuged at 15000 × g, 4 °C, for 30 min to remove cell debris and proteins. The supernatant was collected and proceeded for exosome purification and analysis.

### Transmission electron microscopy (TEM) for exosome

100 μl of freshly filtered-collected aqueous humor samples were ultracentrifuged for 120000 × g, 4 °C, 2 h. The supernatant was discarded so as not to disturb the pellet. The pellet was then reconstituted at 10 μL sterile PBS. The reconstituted liquid was dropped on a nickel-carbon grid (Electron Microscopy Sciences, #CF200-Ni-50, US), and 2.5% glutaraldehyde was dropped on the grid for 1:1 volume for 10 min for fixation. 1% uranyl acetate solution was dropped for gentle flow-thru and left in RT for 2 min for exosome staining. The grid was washed with sterile distilled water 3 times with gentle dropping-flow-thru and let dry. The images were immediately obtained using a transmission electron microscope (H-7000, Hitachi, Japan).

### Exosome purification

100 μL of freshly filtered-collected aqueous humor samples were collected in the new tube, and the exosome was isolated using ExoQuick-TC Ultra for Tissue Culture Media (System Bioscience, #EQULTRA-20TC-1, US), modifying the manufacturer’s protocol to accommodate the smaller sample volume. Briefly, the buffers were used for 1/20 volume than the recommended protocol. The isolated exosomes were ultracentrifuged for 120000 × g, 4 °C for 2 h, and the supernatant was discarded without disturbing the pellet. Pellets were reconstituted or lysed in buffers for the next analyses.

### Exosome size analysis

For the size analysis of purified exosomes, the pellet was reconstituted in sterile DEPC water-based 1 × PBS to create a final volume of 100 μL. The fresh aqueous humor or the purified-reconstituted exosome samples were diluted to 1:3 with sterile PBS. For DLS (Direct Light Scattering) analysis, particle size was measured using Zetasizer (NANO-ZS ZEN3600, Malvern Pananlytical, UK). The analytical report was obtained after performing 10 measurements per each sample for three different samples in the disease group. The concentration was compensated using a dilution factor of 3.00e + 0. Nanoparticle Tracking Analysis (NTA) was performed for individual samples using NanoSight (NS300, Malvern Pananlytical, UK). The traces were recorded for 60 s, 5 times per sample at 25 °C. The concentration was compensated using a dilution factor of 3.00e + 0.

### Exosomal RNA isolation and analysis

For the exosome-purified pellet, 35 μL of QIAzol was added and proceeded for total RNA isolation including both mRNA and miRNA using miRNeasy Micro Kit (Qiagen, #1071023, Germany) following the provided protocol. For mRNA analysis, weverse transcription was performed to synthesize cDNA using PrimeScript 1st strand cDNA synthesis kit (TAKARA, #6110A, JAPAN). Real-time qPCR for STC2 mRNA was performed with BioRad CFX Connect using SsoAdvance Universal Supermix (BioRad, #1725270, USA). For miRNA analysis, reverse transcription was performed with 10.0 ng RNA using miR-184-specific reverse transcription primer and quantified with miR-184 probe-based real-time qPCR using TaqMan MicroRNA Reverse Transcription Kit (Applied Bioscience, #4366596, US), miR-184-specific TaqMan MicroRNA Assay (Applied Bioscience, #4427975, US), and TaqMan Universal Master Mix II (Applied Bioscience, #4440040, US) following the manufacturer’s protocol. Real-time qPCR was performed with BioRad CFX Connect and analyzed with CFX Maestro software.

### Aqueous humor miRNA isolation and analysis

Fresh aqueous humor sample or exosome purified liquid was filtered using a 4 mm RA membrane syringe filter, and filtered aqueous humor was collected. TRIzol LS (Invitrogen, #10296028, US) was added to the filtered aqueous humor following the manufacturer’s protocol. miRNA isolation and miR-184 detection were performed as an exosomal miRNA analysis procedure.

### Human primary retina and RPE-choroid tissue culture

Human primary retina and RPE/choroid tissues were isolated from fresh donor eyes; a 60-year-old female donor without metabolic or ocular disease history. The retina and choroid tissue were separated from the donor eye immediately after cornea buttoning and was radially cut into four leaves. The tissues were cultured in a 60pi culture dish each in a mixture of culture media for eye tissue culture; Exo-free FBS (System Biosciences, #EXO-FBS-250A-1, US) was mixed with serum-free DMEM F/12, Neurobasal Media supplemented with B27, Pericyte Growth Media and Endothelial Growth Media was mixed in adequate proportions for each retina and RPE-choroid and cultured for 36 h.

### Human primary tissue-derived exosomal miRNA analysis

The media soup was collected 36 h after tissue culture. Exosomes from the collected soup and naïve media were purified using ExoQuick-TC Ultra (System Biosciences, #EQULTRA-20TC-1, US) for Tissue Culture Media for the aqueous humor exosome purification, following the provided protocol, and isolated as a pellet by ultracentrifugation.

### Human primary choroidal endothelial cell isolation and culture

Human primary choroidal endothelial cells (hCEC) were isolated from fresh donor eyes aged approximately 30 without metabolic or ocular disease history; 33-year-old female donor and a 31-year-old male donor. The choroid tissue was separated from the donor eye immediately after cornea buttoning and was dissociated into single cells using 1U/mL of Collagenase/Dispase (Roche, #10269638001, Germany) in a shaking incubator (37 °C, 200 rpm, 2 h) and filtered through a 40 μm cell strainer. The CD31-positive endothelial cells were isolated using Dynabeads magnet-based cell isolation system with anti-CD31 Dynabeads (ThermoFisher, #11155D, US) following the manufacturer’s protocol. CD31-positive hCECs were cultured in a 1% gelatin-coated dish with microvascular endothelial cell growth medium-2 (EGM2-MV; LONZA, #CC-3202, Switzerland) in 37 °C, 5% CO2 condition up to passage 2.

### miRNA mimic/inhibitor transfection

miR-184 mimic, inhibitor, and non-targeting scrambled negative control RNAs (Bioneer Co. Ltd, Daejeon, Korea) were used. Cells were transfected with miR-184 mimics, inhibitors, or negative control RNAs using Lipofectamin™ RNAiMAX Transfection reagent (Invitrogen, #13778150), according to the manufacturer’s instructions. Synthetic miRNA oligomers were complexed with the transfection reagent in Opti-MEM, reduced serum medium, and added to cells. Cells were then used for the following assays 24–48 h after transfection.

### STC2 siRNA transfection

STC2 siRNA and scrambled siRNA for negative control (Bioneer Co. Ltd, Daejeon, Korea) were used to induce STC2 knock-down. Cells were transfected with siRNAs using Lipofectamin™ 3000 Transfection reagent (Invitrogen, # L3000008), according to the manufacturer’s instructions. Synthetic siRNA oligomers were complexed with the transfection reagent in Opti-MEM, reduced serum medium, and added to cells. Cells were then used for the following assays 24 h-36 h after transfection.

### Total RNA real-time quantitative PCR (qPCR)

Total RNA was extracted from the cultured cells by TRIzol Reagent (Invitrogen, Carlsbad, CA, USA) per the manufacturer’s instructions. Reverse transcription was performed to synthesize cDNA using PrimeScript 1st strand cDNA synthesis kit (TAKARA, #6110A, JAPAN). Real-time qPCR was performed with BioRad CFX Connect using iQ SYBR Green Supermix (BioRad, #1708880, USA) and analyzed with CFX Maestro software.

The qPCR primers and their sequences are listed in Additional file 3: Table S1.

### 3D tube formation assay

hCEC transfected with miRNA oligo (miR-184 mimic, inhibitor or negative controls) and siRNA oligo (STC2 siRNA or negative control) were seeded in a Matrigel^®^ Matrix (Corning, NY, USA) coated 96-well plate at 2 × 104 cells/well density. Cells were incubated at 37 °C, 5% CO2 for 4 h, and imaged at 4 h after seeding. Tube formation parameters were analyzed using WimTube online software (Onimagin, Córdoba, Spain).

### In vitro wound healing assay

hCEC transfected with miRNA oligo (miR-184 mimic, inhibitor or negative controls) and siRNA oligo (STC2 siRNA or negative control) were seeded at a concentration of 2 × 10^5^ cells/well at a 24-well plate to reach the confluent monolayer in 24 h. Cell monolayers were scraped with a 200 μL pipette tip and incubated at 37 °C, 5% CO2. Since the doubling time of the endothelial cells are more than 17 h, images of the cells were taken immediately after scraping and at 12 h to analyze the wound closure by cell migration [59]. The closed wound spaces were quantified and normalized to the originally generated wound spaces.

### In vitro 3D microfluidic angiogenic assay

Microfluidic plastic chips and chip holders to maintain chip humidity were purchased from AIM Biotech company (AIM Biotech, Singapore). Collagen type I solution (Corning, NY, USA) at 2 mg/mL was gently pipetted into the gel-filling inlet of the devices and polymerized for 30 min at 37 °C and 5% CO2 assembled in the humidified chip holder. 1/10 diluted human plasma fibronectin (Sigma-Aldrich, #F0895, US) was injected into the microchannels, and the device was incubated for 1 h at 37 °C and 5% CO2 in the humidified chip holder. hCEC transfected with miR-184 mimic/inhibitor or negative controls were seeded in one of the fluidic channels at 6 × 10^4^ cells/channel. Twenty-four hours after seeding, recombinant Human VEGF165 (Peprotech, #100–20, Rocky Hill, NJ, USA) at a final concentration of 40 and 20 ng/mL according to the manufacturer’s instructions were added to the growth media to generate a VEGF gradient, as confirmed in our previous study [60]. Cells were incubated at 37 °C and 5% CO2, and the medium with VEGF was changed every 24 h. Cell sprouting was monitored and imaged every 24 h. The number of tip cells/mm was counted from the acquired image for sprouting angiogenic property parameters.

### STC2 Western blot analysis

Cells transfected with miR-184 mimic/inhibitor or negative controls for 48 h were washed with three changes of PBS. After removing PBS, cells were immediately frozen with liquid N_2_. Cold 1 × Pierce RIPA Buffer (Thermo Scientific, #89,900, US) containing 1 × HALT Phosphatase Inhibitor Cocktail (Thermo Scientific, #78420, US) and 1 × HALT Phosphatase Inhibitor Cocktail (Thermo Scientific, #78430, US) were added immediately for cell lysis. Cells were scraped from the plastic surface and collected in Protein LoBind Tubes. After 10 min incubation on ice, cell lysates were centrifuged (13000 g, 4 °C, 30 min), and supernatants containing proteins were acquired. Protein concentration was measured using the Pierce BCA Assay Kit (Thermo Scientific, #23227, US) following the manufacturer’s protocol. Samples were blotted to the NC membrane (GE Healthcare Life Science, #10600114, Germany) after electrophoresis. The blotted membrane was then blocked using EveryBlot Blocking Buffer (BioRad, #12010020, US). The antibodies used for detection were rabbit anti-STC-2 (Abcam, #ab63057, US), and mouse anti-α-tubulin (Santa Cruz, #SC-5286, US), goat-anti-rabbit-HRP (Genetex, #GTX213110-01, US), and goat-anti-mouse-HRP(Genetex, #GTX213111-01, US) diluted to the manufacturer’s recommended concentration.

### Statistical analysis

The representative values are presented as mean ± standard deviation (SD). Statistical significance was analyzed with Welch’s *t*-test and Student’s *t*-test and defined as * *P* < *0.05*, **P < 0.01, and *** P < *0.001*. Statistical analyses were performed using R × 64 v4.1.1.

### Image analysis

The microscopic images for cell functional assay analysis were imaged using an inverted microscope (Olympus IX70, Japan) using DP controller software. The images were quantified and analyzed using Java-based imaging software (ImageJ, v.1.52p, in the public domain at http://rsb.info.nih.gov/ij; National Institutes of Health (NIH), Bethesda, MD, USA) [26].

## Supplementary Information

**Additional file 1: Figure S1**. Validation of target gene downregulations by miR-184 mRNA expression of miR-184 target genes in miR-184 mimic transfected hCEC. The data are arranged in ascending order of expression level. *STC2* mRNA (demarked in the blue box) is significantly reduced when miR-184 mimics are transfected. The graph values are represented as mean ± standard deviation. The y-axis shows log_2_ fold change compared with the reference control (ZIC4 expression of N.C treated). Statistical significance indicated as *P<0.05, **P<0.01, ***P<0.001.

**Additional file 2: Data 1**. Raw value of the individual expression level of miR-184 and *STC2 *of the controls and patients with CSC

**Additional file 3: Table S1**. Sequences of qPCR primers and miRNA mimic/inhibitor.

## Data Availability

Raw and processed data have been deposited at GEO (https://www.ncbi.nlm.nih.gov/geo/) (accession no. GSE227142). All relevant data area available upon request (j.lee@amc.seoul.kr).

## Abbreviations

AH: Aqueous humor
AMD: Age-related macular degeneration
CEC: Choroidal endothelial cell
hCEC: Primary cultured-human choroidal endothelial cells
CNS: Central nervous system
CRT: Central retinal thickness
CSC: Central serous chorioretinopathy
-R: Anti-VEGF treatment responder
-NR: Anti-VEGF treatment non-responder
DLS: Dynamic light scattering
IL-6: Interleukin 6
IL-8: Interleukin 8
miRNA: MicroRNA
NGS: Next-generation sequencing
NTA: Nanoparticle tracking analysis
OCT: Optical coherence tomography
qPCR: Quantitative polymerase chain reaction, real-time PCR
RPE: Retinal pigment epithelium
RNA: Ribonucleic acid
SFChT: Subfoveal choroidal thickness
SRF: Subretinal fluid
STC1: Stanninocalcin 1
STC2: Stanninocalcin 2
TEM: Transmission electron microscopy
VEGF: Vascular endothelial growth factor
