# Supplementary figures and images for: Exosomal miR-184 in the aqueous humor of patients with central serous chorioretinopathy: a potential diagnostic and prognostic biomarker
Source: J Nanobiotechnology. 2023 Jul 28;21:242. doi: 10.1186/s12951-023-02019-6 (PMC10375666; doi:10.1186/s12951-023-02019-6)

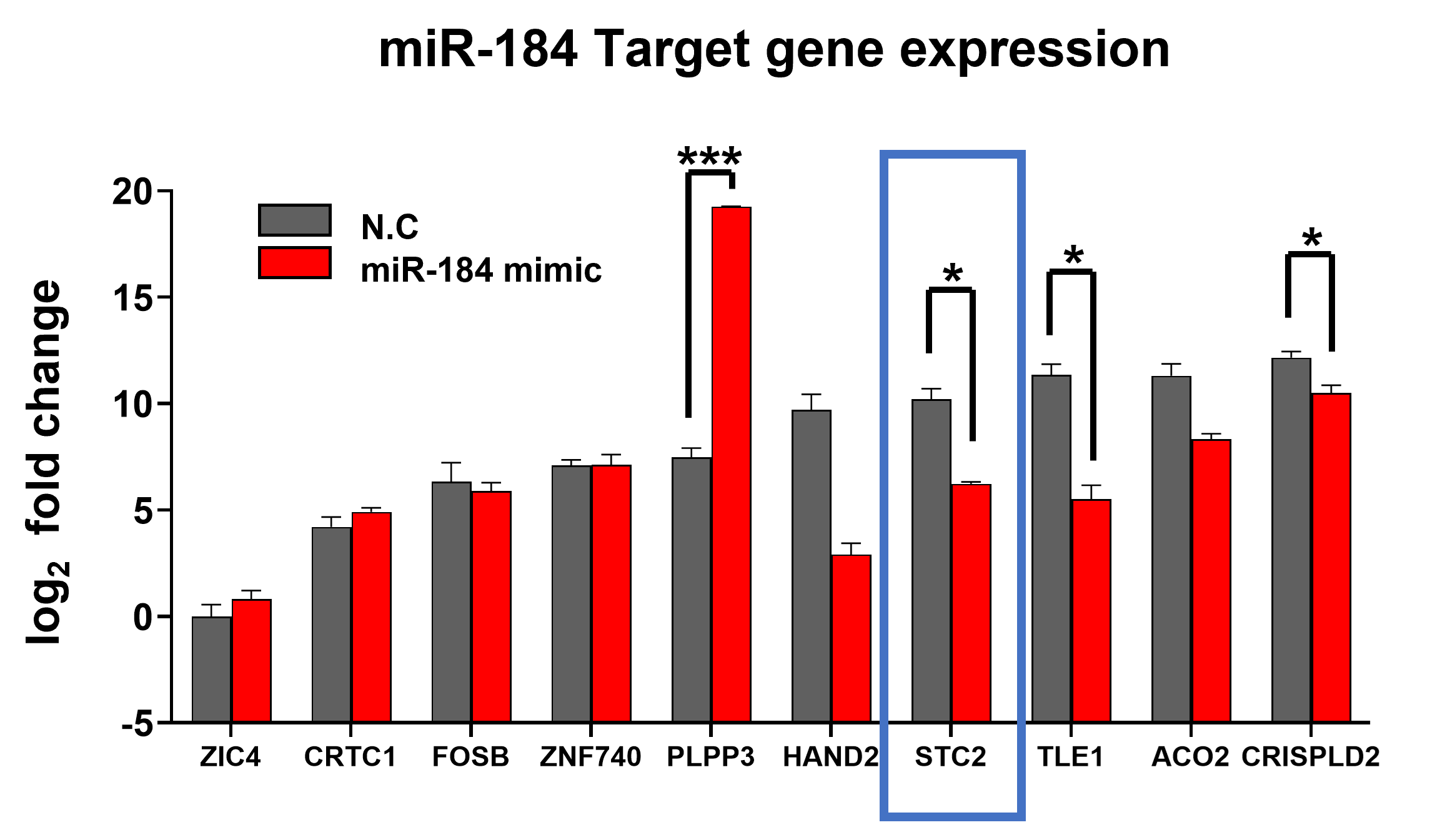

Supplement: Supplementary file 1 — Additional file 1: Figure S1. Validation of target gene downregulations by miR-184 mRNA expression of miR-184 target genes in miR-184 mimic transfected hCEC. The data are arranged in ascending order of expression level. STC2 mRNA (demarked in the blue box) is significantly reduced when miR-184 mimics are transfected. The graph values are represented as mean ± standard deviation. The y-axis shows log2 fold change compared with the reference control (ZIC4 expression of N.C treated). Statistical significance indicated as *P<0.05, **P<0.01, ***P<0.001. [file 12951_2023_2019_MOESM1_ESM.tif]
