# Supplementary material for: Exosomal miR-184 in the aqueous humor of patients with central serous chorioretinopathy: a potential diagnostic and prognostic biomarker
Source: J Nanobiotechnology. 2023 Jul 28;21:242. doi: 10.1186/s12951-023-02019-6 (PMC10375666; doi:10.1186/s12951-023-02019-6)
Supplement: Supplementary file 3 — Additional file 3: Table S1. Sequences of qPCR primers and miRNA mimic/inhibitor. [file 12951_2023_2019_MOESM3_ESM.docx]

Additional file 1: Table S1. qPCR primer sequence and miR-184 mimic/inhibitor sequence

|  | Gene | qPCR Primer Sequence (5’->3’) | |
| --- | --- | --- | --- |
| Primer | GAPDH | F | CCTCCAAGGAGTAAGACCCC |
|  |  | R | AGGGGTCTACATGGCAACTG |
|  | CRTC1 | F | TTCCAGACCCCCTTCCAATC |
|  |  | R | CTGGGACCCACTGCTAAAGG |
|  | ZNF740 | F | ATGGCTCAGGCAAGTCTCCT |
|  |  | R | TTTTCGGTGACCCTGACTCG |
|  | ZIC4 | F | GCTTTTGGAGGGTTGCACTT |
|  |  | R | TTCTTACGGTCGCTGCTGTT |
|  | PLPP3 | F | AGACAAGCACCATCAAGCCT |
|  |  | R | GCCACGTAGGGGTTCTGAAT |
|  | TLE1 | F | TCACTATCCCGGAGTCCCTG |
|  |  | R | TGCCCGATGATGGCATTCAA |
|  | ACO2 | F | CATTGAAAACGGCAAGGCCA |
|  |  | R | CTCGCCGTAGTTCTCGTCTC |
|  | FOSB | F | AACCAGCTACTCCACACCAG |
|  |  | R | ACTGATCTGTCTCCGTCTCCT |
|  | HAND2 | F | CTACTTCCATGGCTGGCTCA |
|  |  | R | GTAGGCGATGTAGCTGGTGG |
|  | STC2 | F | TGAAATGTAAGGCCCACGCT |
|  |  | R | CGAGGTGCAGAAGCTCAAGA |
|  | CRISPLD2 | F | ACCCACCAAGCCCAAGAAAA |
|  |  | R | ATATCCACCAGGCCTCCCTT |
| miRNA | hsa-miR-184-mimic |  | UGG ACG GAG AAC UGA UAA GGG U |
|  | hsa-miR-184-inhibitor |  | UGG ACG GAG AAC UGA UAA GGG U |
